# 4327 Structural Neural Correlates of Social Functioning in First Episode Psychosis and Malleability in Response to Targeted Cognitive Training

**DOI:** 10.1017/cts.2020.148

**Published:** 2020-07-29

**Authors:** Kathleen Miley, Fang Yu, Ian Ramsay, Sophia Vinogradov

**Affiliations:** 1 University of Minnesota CTSI

## Abstract

OBJECTIVES/GOALS: Development of interventions that improve social functioning (SF) in first episode psychosis (FEP) is hindered by a poor understanding of the neural mechanisms underlying SF deficits. This research aims to identify neural correlates of social functioning in FEP, and to evaluate whether this substrate is malleable in response to cognitive training. METHODS/STUDY POPULATION: This is a secondary data-analysis of participants in an ongoing randomized clinical trial investigating whether 12 weeks of targeted cognitive training is neuroprotective in FEP, versus treatment as usual. Baseline and post-training assessments include a brain MRI, three measures of SF, and a neurocognitive battery. Healthy controls complete MRI only. Differences in cortical thickness (CTh) and gray matter volume (GMV) in regions of interest between FEP and controls will be determined with ANCOVA. Multiple linear regression will be used to determine the relationship between neural substrate and SF in FEP. Linear mixed models will be used to examine the relationship between change in CTh and GMV and change in SF. Data collection is ongoing for this study. RESULTS/ANTICIPATED RESULTS: In preliminary data including 12 FEP and 9 healthy controls, FEP demonstrated cortical loss in the right superior frontal cortex and the right isthmus-posterior cingulate. Greater cortical thickness in the posterior cingulate cortex was associated with better social functioning across multiple measures when controlling for global cognition. Gray matter volume in the parahippocampal gyrus was also associated with better social functioning. Preliminary results evaluating whether targeted cognitive training is neuroprotective in these regions of interest in a manner that is associated with improved social functioning will also be presented. DISCUSSION/SIGNIFICANCE OF IMPACT: Preliminary results link the posterior cingulate and parahippocampal gyrus to SF in FEP. Further research will investigate the contribution of changes in these brain regions to improved SF. The identification of biological treatment targets for SF may lead to development and optimization of novel interventions to alleviate SF deficits in FEP.

